# A human-centered safe robot reinforcement learning framework with interactive behaviors

**DOI:** 10.3389/fnbot.2023.1280341

**Published:** 2023-11-09

**Authors:** Shangding Gu, Alap Kshirsagar, Yali Du, Guang Chen, Jan Peters, Alois Knoll

**Affiliations:** ^1^Department of Computer Science, Technical University of Munich, Munich, Germany; ^2^Department of Computer Science, Technical University of Darmstadt, Darmstadt, Germany; ^3^Department of Informatics, King's College London, London, United Kingdom; ^4^College of Electronic and Information Engineering, Tongji University, Shanghai, China

**Keywords:** interactive behaviors, safe exploration, value alignment, safe collaboration, bi-direction information

## Abstract

Deployment of Reinforcement Learning (RL) algorithms for robotics applications in the real world requires ensuring the safety of the robot and its environment. Safe Robot RL (SRRL) is a crucial step toward achieving human-robot coexistence. In this paper, we envision a human-centered SRRL framework consisting of three stages: safe exploration, safety value alignment, and safe collaboration. We examine the research gaps in these areas and propose to leverage interactive behaviors for SRRL. Interactive behaviors enable bi-directional information transfer between humans and robots, such as conversational robot ChatGPT. We argue that interactive behaviors need further attention from the SRRL community. We discuss four open challenges related to the robustness, efficiency, transparency, and adaptability of SRRL with interactive behaviors.

## 1. Introduction

Deep learning has shown impressive performance in recent years (LeCun et al., 2015; Wang et al., 2022; Liu et al., 2023; Zhao and Lv, 2023). By leveraging deep learning, Reinforcement Learning (RL) has achieved remarkable successes in many scenarios and superhuman performance in some challenging tasks (Gu et al., 2022b, 2023), e.g., autonomous driving (Gu et al., 2022a), recommender system (Zhao et al., 2021), robotics (Brunke et al., 2021), games (Silver et al., 2018; Du et al., 2019; Han et al., 2019), and finance (Tamar et al., 2012). Most RL methods aim to maximize reward performance without considering safety constraints. However, safety is critical when deploying RL in real-world applications, especially in robotics. In Robot RL (RRL), a robot interacts with static or dynamic environments to learn the probability of better actions. When humans are also part of the robot's environment, ensuring their safety is crucial. This paper proposes a framework to achieve Safe Robot RL (SRRL) by leveraging interactive behaviors.

**Figure 1:**
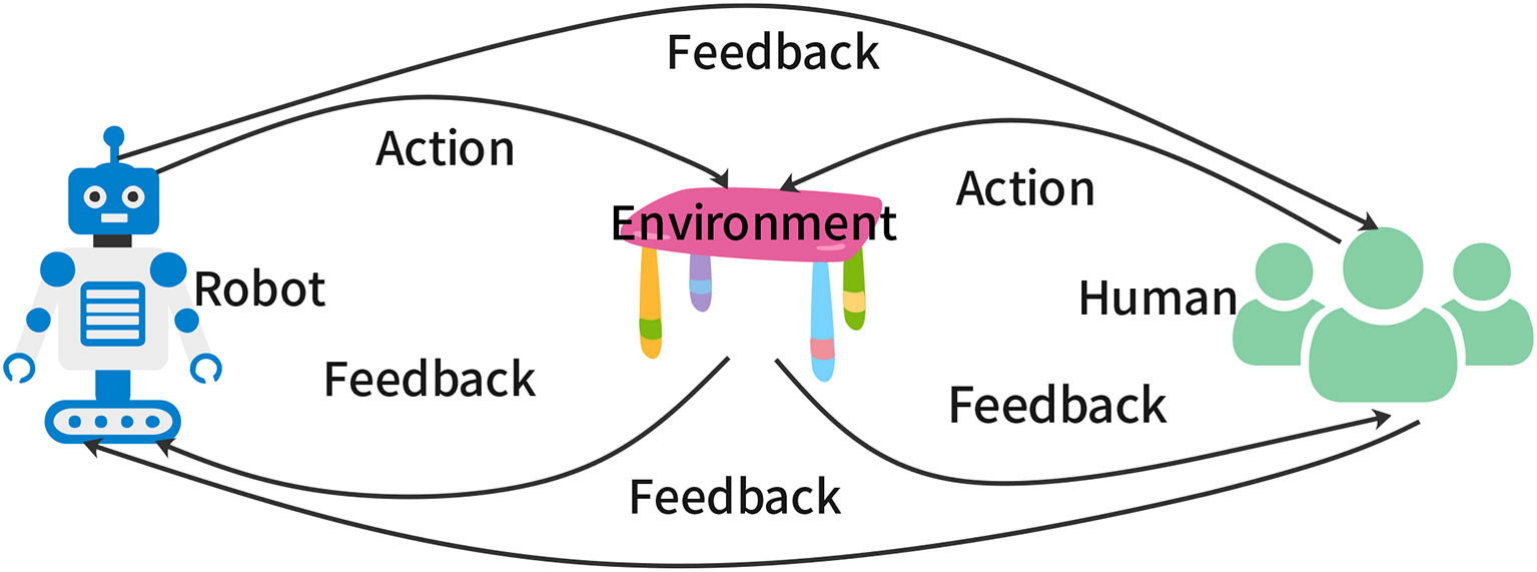
Schematic of interactive behaviors.

Interactive behaviors are behaviors that can mutually influence the interacting elements. Interaction is everywhere in human life (Kong et al., 2018), and agent-environment interaction is the basis of RL (Sutton and Barto, 2018). When humans and robots act in a shared environment, their actions can be influenced by each other through interactive behaviors (Thomaz and Breazeal, 2006; Knox and Stone, 2009; MacGlashan et al., 2017; Kazantzidis et al., 2022; Lou et al., 2023). For example, a robot navigating in a public space can plan its path to avoid collisions with pedestrians and provide signals to pedestrians to move aside. The pedestrians can also plan their path to avoid collisions with the robot and provide signals to the robot to move aside. Figure 1 shows a schematic of interactive behaviors consisting of three elements: robots, environment, and humans. The outer loop consists of feedback or signals from the robots to the humans and vice versa. The two inner loops consist of actions from robots and humans in the environment and feedback or rewards from the environment to robots and humans. Here, we use the term “feedback” to mean any type of information transfer between the two interacting elements.

Interactive behaviors can lead to better SRRL by enabling bi-directional information transfer between the interacting elements. It is a core technology to improve the dialogue performance of a Large Language Model (LLM), e.g., ChatGPT (OpenAI, 2023) by leveraging interactive behaviors. For example, in ChatGPT, on the one hand, high-quality data from human feedback is collected to design the reward model (Stiennon et al., 2020; Gao et al., 2022). On the other hand, after having human feedback, the agent model will be trained to align human values, and then give safe feedback to humans. In most conventional SRRL approaches, there is no human interaction, e.g., CPO (Achiam et al., 2017) and ATACOM (Liu P. et al., 2022). With interactive behaviors, the robot can learn about human behaviors and convey its features and decision-making processes to humans. Those approaches that consider the human-in-the-loop of the robot's learning process do not utilize feedback from the robot learner to the human teacher.

In this paper, we investigate interactive behaviors to achieve three stages of human-centered SRRL: “safe exploration,” “safety value alignment,” and “safe human-robot collaboration.” In the “safe exploration” stage, the robot must explore the unknown state space while preserving safety. In the “safety value alignment” stage, the robot has to align its intentions with the humans. Finally, in the “collaboration” stage, the robot should contribute toward achieving shared goals with humans.

## 2. Related work on safe robot-reinforcement learning

Safe robot learning has received substantial attention over the last few decades (Turchetta et al., 2019; Baumann et al., 2021; Kroemer et al., 2021; Marco et al., 2021; Kaushik et al., 2022). SRRL methods focus on robot action and state optimization and modeling to ensure the safety of robot learning. For instance, Gaussian models are used to model the safe state space (Akametalu et al., 2014; Sui et al., 2015, 2018; Berkenkamp et al., 2016; Turchetta et al., 2016; Wachi et al., 2018); formal methods are leveraged to verify safe action and state space (Fulton and Platzer, 2018; Kochdumper et al., 2022; Yu et al., 2022); control theory is applied to search safe action space (Chow et al., 2018, 2019; Koller et al., 2018; Li and Belta, 2019; Marvi and Kiumarsi, 2021).

Akametalu et al. (2014) introduced a reachability-based method to learn system dynamics by using Gaussian models, in which the agent can adaptively learn unknown system dynamics and the maximal safe set. Berkenkamp et al. (2016) proposed a region of attraction method to guarantee safe state space based on Gaussian processes and Bayesian optimization. Although the method provided a theoretical analysis for safe robot learning, the assumptions in the study are quite strong and may not be applicable in practical environments. Sui et al. (2015) present a safe exploration method considering noise evaluations, in which the safety is ensured with a high likelihood via a Gaussian process confidence bound. Moreover, the sample complexity and convergence of the method are analyzed, and real-world applications, such as movie recommendations and therapeutic spinal cord stimulation, are used to test how well the system works. Nonetheless, they considered the safe exploration as a bandit setting without any constraints (Turchetta et al., 2016). Turchetta et al. (2016) developed an algorithm in which Gaussian processes are leveraged to model the safe constraints. However, a set of starting safe states is required from which the agent can begin to explore in the work of Sui et al. (2015) and Turchetta et al. (2016).

Some recent works have investigated the application of formal methods for SRRL. Fulton and Platzer (2018) used formal verification to check the correctness of the state transition model and select safe action during RL. They allow unsafe actions if the model is incorrect. Researchers have proposed three types of provably safe RL methods for hard safety (Krasowski et al., 2022): action mask, action replacement, and action projection. Action masking approaches apply a safety layer to restrict the agent's actions to safe actions only (Krasowski et al., 2020). Action replacement approaches replace unsafe actions with safe actions (Hunt et al., 2021). Action projection methods project the unsafe actions to close safe actions (Kochdumper et al., 2022).

Control theory based SRRL approaches have utilized Lyapunov functions and Model Predictive Control (MPC). Chow et al. (2018, 2019) introduced Lyapunov functions based on discrete and continuous control methods for the global safety of behavior policy in RL. However, designing Lyapunov functions for different environments may be difficult, and the requirement of a baseline policy may be challenging to satisfy during real-world applications. Koller et al. (2018) proposed an MPC based method to ensure safe exploration using a statistical model of the system. Marvi and Kiumarsi (2021) introduced a control barrier function method for safe off-policy robot learning without needing to have a thorough understanding of system dynamics, in which the cost functions are augmented by a control barrier function.

**Figure 2:**
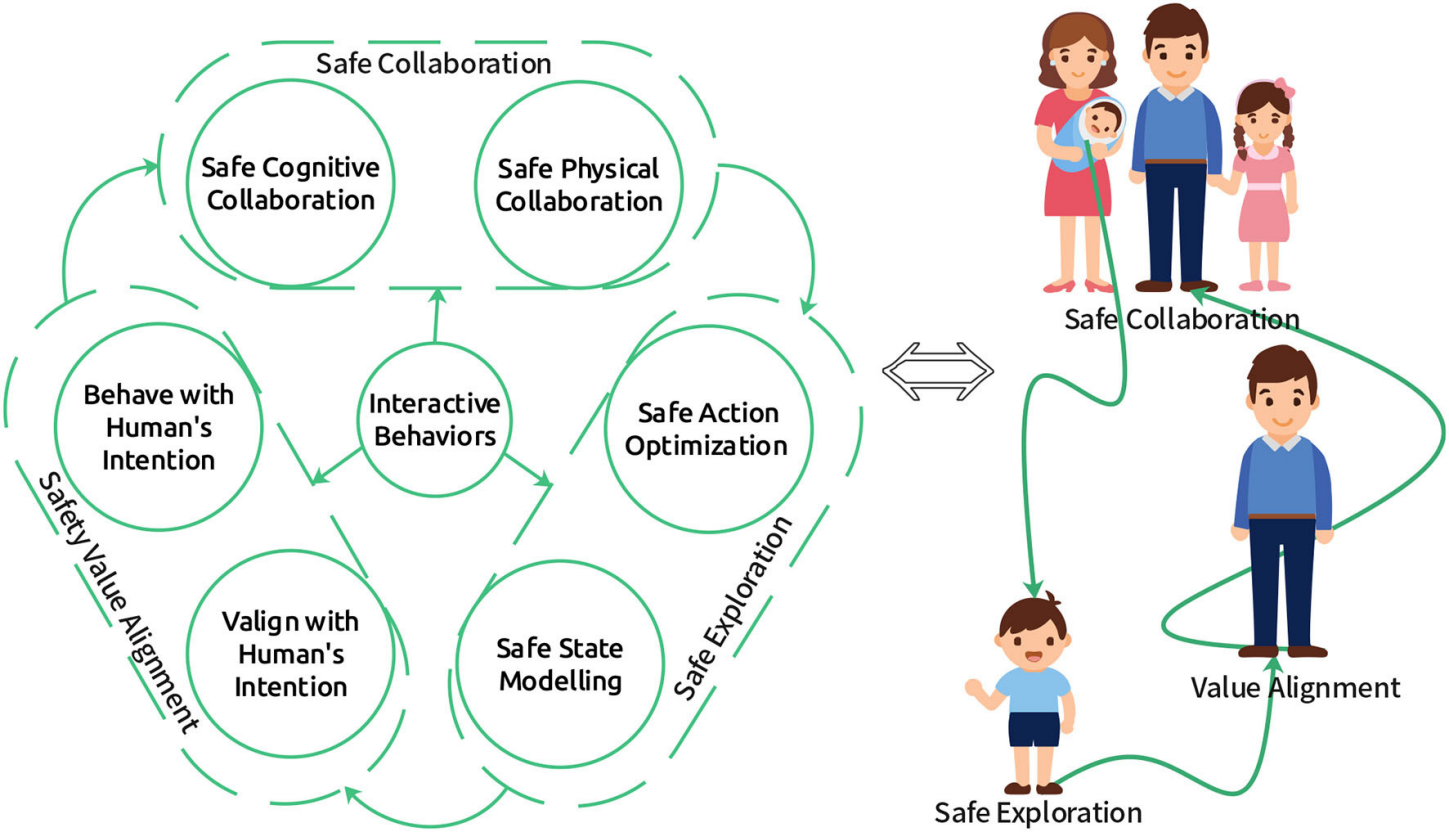
Three stages of human-centered SRRL. When a newborn infant enters a new environment, if it wants to survive in the new environment, it must first learn about the environment via interactive behaviors (**Safe Exploration**), e.g., bi-directional feedback with its parents to learn how to walk. Then it should find something helpful to survive better via interactive behaviors (**Value Alignment**), e.g., bi-directional feedback with its teachers to learn human values. Finally, it has to collaborate with others to maximize the society's reward via interactive behaviors (**Safe Collaboration**), e.g., bi-directional feedback with its collaborators to create new things.

Most of the above methods do not consider human-robot interaction. There are some prior works that investigate human-centered SRRL. For instance, Kazantzidis et al. (2022) introduced a mechanism to ensure safety during exploration by harnessing human preferences. Reddy et al. (2020) present a method to learn the model of human objectives by leveraging human feedback based on hypothetical behaviors, and then the model can be used to ensure the safety of robot learning. Saunders et al. (2018) try to guarantee reinforcement learning safety by human interventions, where human interventions are learned through a supervised learning model. However, most of these works do not consider the mutual influence of humans and robots in shared environments. Thus, interactive behaviors between humans and robots that leverage bi-directional information transfer, as shown in Figure 2, still need further investigation to ensure SRRL.

## 3. Human-centered safe robot reinforcement learning framework

Our proposed human-centered SRRL framework, as shown in Figure 2, consists of three stages: safe exploration, safety value alignment, and safe collaboration. When a robot enters a new environment, it must explore it through safe action optimization and state modeling. Then, the robot has to learn the safety value alignment from the human-robot interaction. Finally, the robot should be able to achieve safe collaboration with humans. Interactive behaviors can help successfully achieve each of these three stages.

### 3.1. Safe exploration

A robot should explore and find helpful information when entering a new environment. During the exploration, the robot must ensure its and the environment's (including other agents') safety. Therefore, the first stage of human-centered SRRL, safe exploration, focuses on exploring new environments and getting information about the environment model through safe actions. Uncertainty about the environment makes it difficult to determine safe actions. For example, newborn infants sometimes end up putting harmful objects in their mouths during oral exploration. It is challenging to ensure safe actions using conventional RL methods that learn new skills through trial and error. Especially with model-free RL, a robot cannot avoid a destructive action unless it has already tried it (or a similar action; Saunders et al., 2018).

Several methods have been proposed to address the challenge of safe exploration for RL (Garcia and Fernández, 2012; Liu et al., 2020; Bharadhwaj et al., 2021; Turchetta, 2021; Xiong and Diao, 2021; Liu P. et al., 2022; Liu Z. et al., 2022). For example, Garcia and Fernández (2012) introduced a safe exploration method, in which a predefined baseline policy is required, and the baseline policy is approximated by behavioral cloning methods (Anderson et al., 2000). Nonetheless, derived from the method, it would be hard to search for the optimal exploration policy, and the capabilities of the baseline policy could severely limit the method's performance. A conservative safety critic (Bharadhwaj et al., 2021) is proposed to guarantee safety with high probability during robot exploration processes. Garcia and Fernández (2012) introduced a smoother risk function for safe robot exploration, which can achieve monotonic reward improvement and ensure safety. This method needs a predefined baseline policy to explore the safe space. Liu P. et al. (2022) developed a safe exploration method for robot learning by constructing a constrained manifold. This method can guarantee safety for robot exploration using model-free RL. However, it requires an accurate robot model or a perfect tracking controller, which may hinder their method's real-world applications.

Interactive behaviors can be used to transfer expert knowledge from the human to the robot for safe exploration, just like infants are safeguarded by their parents. Human intervention has been investigated to avoid catastrophes in RL (Saunders et al., 2018), and human feedback can be a reference for RL to ensure robot safety during safe exploration (Frye and Feige, 2019). However, the amount of human labor required for complex real-world applications is prohibitive. The bi-directional feedback in interactive behaviors can reduce the human-time cost during safe exploration. On the one hand, the robot can actively query the human and provide explanations of its behavior. On the other hand, the human can also actively query the robot to learn about the robot's model, in addition to intervening for safe exploration.

### 3.2. Safety value alignment

In the second stage of safe robot learning, to train a robot to perform a task safely, we need to evaluate how well it performs in terms of its performance on safety. Whether it is a form of costs, rewards, or labels, we need some form of signals to guide the safety policies for robot learning. In some scenarios, the safety performance can be evaluated automatically, such as bumping into other vehicles of autonomous driving or breaking the arms in robot manipulation. In these cases, training signals can be straightforwardly defined for safe robot learning. Furthermore, to facilitate the safe deployment of agents in real-world tasks, agents also need to be compatible with users' ethical judgment. Taking automated vehicles as an example, should one autonomous vehicle cut in line to maximize its reward in achieving a goal? Instead of maximizing only the reward, the agents need the capacity to abide by human moral values, which is essential but lacks effort.

Current robot learning algorithms rely on humans to state these training signals (Gu et al., 2022b; Yuan et al., 2022) and assume that humans understand the dangers. For example, imitation learning inferences a reward function from human demonstrations; preference-based learning guides the robot based on human judgments. These classes of tasks involve “human” training signals. The related problem of how to align has been discussed in earlier literature (Christiano et al., 2018; Leike et al., 2018; Kazantzidis et al., 2022; Liu R. et al., 2022) on how to align agents with user intentions, in which meaningful training signals can be hard to obtain, due to the unpredictable long-term effect of the behaviors, or potential influence to other agents and environments in large multi-agent systems.

Designing AI agents that can achieve arbitrary objectives, such as minimizing some cost or penalties, can be deficient in that the systems are intrinsically unpredictable and might result in negative and irreversible outcomes for humans. In the context of interactive learning, we consider how a robot can behave safely or align with the user's intentions whilst maintaining safety under interactive behaviors with humans, and we frame this as the **safety value alignment** problem: *how to create robots that behave safely and align with the human's intentions?*

Interactive behaviors allow agents to infer human values. While agents infer human values from their feedback, bi-directional feedback enables the agent to explain its decision-making process. One early attempt (Yuan et al., 2022) studied bi-directional communication in tabular-based navigation tasks without considering more practical scenarios. The next step aims to study the generalization of such results to large-scale problems via more efficient algorithms.

### 3.3. Safe human-robot collaboration

The third stage of our safe-robot learning framework aims to accomplish safe physical and cognitive collaboration between robots and humans. Collaboration has enabled humans to achieve great evolutionary success. Therefore, safe human-robot collaboration is essential for successful human-robot co-existence.

The four main categories of human-robot collaboration tasks explored in the literature are collaborative assembly, object handling, object handovers, and collaborative manufacturing (Semeraro et al., 2023). RL has been used for tuning impedance controllers in physical human-robot collaboration tasks such as lifting objects (Roveda et al., 2020) and guided trajectory following (Modares et al., 2015). However, these works evaluated the controllers in simplified scenarios and did not evaluate the generalizability of the learned policies. RL has also been used for performing robot-to-human object handovers (Kupcsik et al., 2018) and human-to-robot object handovers (Chang et al., 2022). Nevertheless, in some cases, the spatial generalizability of learned policies is low (Kshirsagar et al., 2021). Ghadirzadeh et al. (2020) used deep q-learning to generate proactive robot actions in a human-robot collaborative packaging task. However, they only evaluated a specific task scenario with a highly engineered reward function. Also, they did not test the trained policy in the real world and for different human participants than the training set.

Deep RL methods have been applied in real-world learning scenarios for tasks like quadrupedal walking, grasping objects, and varied manipulation skills (Ibarz et al., 2021). One of the desired features of these works is the ability to perform training with little or no human involvement. However, scenarios of human-robot collaboration typically involve multiple humans in the robot's learning process. Multi-agent RL methods such as self-play or population-play do not perform very well with human partners (Carroll et al., 2019). One proposed solution called Fictitious Co-Play (FCP) involves training with a population of self-play agents and their past checkpoints taken throughout training (Strouse et al., 2021). However, FCP was evaluated only in a virtual game environment.

Interactive RL (IRL) approaches involve a human-in-the-loop to guide the robot's RL process. IRL has been applied to various human-computer interaction scenarios (Arzate Cruz and Igarashi, 2020). In addition, human social feedback in the form of evaluation, advice, or instruction has also been utilized for several robot RL tasks (Lin et al., 2020). However, more research is needed toward utilizing IRL for safe human-robot collaboration. Also, while some works have explored non-verbal cues to express the robot's uncertainty during the learning process (Matarese et al., 2021), most existing IRL approaches do not involve feedback from robots to humans. As depicted in Figure 2, evaluative feedback from robots to humans could help improve human-robot collaboration.

## 4. Open challenges

In this section, we describe four key open challenges toward utilizing interactive behaviors for SRRL. These open challenges are related to the robustness, efficiency, transparency, and adaptability of SRRL.

1. How can the robot learn robust behaviors with potential human adversaries?
2. How to improve data efficiency of SRRL for effective utilization of interactive behaviors?
3. How to design “transparent” user interfaces for interactive behaviors?
4. How to enhance adaptability of SRRL for handling multiple scenarios of interactive behaviors?

The first challenge is to achieve robust SRRL with respect to unintentional or intentionally erroneous human conduct in interactive behaviors. In existing SRRL methods, it is often neglected that humans might misstate the safety signals. Also, due to the potential involvement of multiple humans with different values, robots need to learn to strike a balance between them. In the extreme case, adversaries may intentionally state their signal to mislead the training of robots to achieve malicious objectives. Training robust agents against such malicious users needs further research. Adversarial training may be useful to ensure safety in such scenarios (Meng et al., 2022). However, adversarial training is not yet ready for real-world robot learning (Lechner et al., 2021).

The second challenge is to improve the data efficiency of SRRL, given that real-world interactive behaviors are expensive. Data efficiency can determine how quickly robots learn new skills and adapt to new environments and how effectively interactive behaviors can be utilized in the learning process. The success of machine learning can be attributed to the availability of large datasets and simulation environments. Therefore, one possible solution to reduce the need for real-world interactive data is to build large datasets or simulations of interactive behaviors. For example, Lee et al. (2022) present a mixed reality (MR) framework in which humans can interact with virtual robots in virtual or augmented reality (VR/AR) environments. It can serve as a platform for collecting data in various human-robot interaction and collaboration scenarios. However, this framework suffers from the limiting aspects of MR, such as the inconvenience of wearable interfaces, motion sickness, and fatigue. Improvements in MR technology will be crucial for the widespread use of such MR environments.

The third challenge is to maintain transparency during SRRL. Transparency is important for the effective utilization of interactive behaviors. MacGlashan et al. (2017) provide empirical results to demonstrate human feedback and robot policy can be interactively influenced by each other, and indicate that the assumption is that the feedback from a human is independent of the robot's current policy, may be incorrect. Transparency can be achieved in the context of interactive behaviors by explaining the robot's decision-making process to the human and exposing the robot's internal state. Several solutions have been proposed to improve the explainability of robot RL (Hayes and Shah, 2017; MacGlashan et al., 2017; van der Waa et al., 2018; Likmeta et al., 2020; Atakishiyev et al., 2021a,b; Matarese et al., 2021). For example, Likmeta et al. (2020) introduced an interpretable rule-based controller for the transparency of RL in transportation applications. Nonetheless, the policy that the method provides may be too conservative, since it severely depends on the restricted rules. Matarese et al. (2021) present a method to improve robot behaviors' transparency to human users by leveraging emotional-behavior feedback based on robot learning progress. However, further research is needed toward communicating the robot's internal state. Non-verbal communication in the form of gazes and gestures can be leveraged to communicate the robot's internal state. For example, if the robot is uncertain about its decisions, it can show hesitation gestures.

The fourth challenge is to enhance the adaptability of safe robot learning to handle a variety of settings involving interactive behaviors. Particularly, a robot can encounter different environments. For instance, completely trusted environments, in which the environment information is known and the environment is stable; **U**nstable environments for which environment information is known; **U**ncertain environments, where the information about the environment is uncertain and partial; **U**nknown environments, in which the environment information is completely unknown. Safety can be ensured in trusted environments, e.g., robots can safely grasp an object in a static environment. However, in “3U” environments, ensuring SRRL is challenging. For instance, during sim-to-real transfer, the discrepancies between the simulation models and real-world models are inevitable (Mitsch and Platzer, 2016), and the real-world environments are replete with uncertain disturbances and unknown information, e.g., in a multi-agent system, guaranteeing each agent's safety may be difficult. Some works provide a potential direction to ensure multi-robot learning safety in unstable environments, for example, Multi-Agent Constrained Policy Optimization and Multi-Agent Proximal Policy Optimization Lagrangian (Gu et al., 2023). Nevertheless, the exploration involving interactive behaviors between agents and environments can be intricate and time-intensive. The incorporation of human insights and value alignment in the exploratory phase is instrumental in enhancing the adaptability of these agents within a human-in-the-loop learning system. As we envisage future trajectories of research, a salient focus is the attainment of SRRL characterized by interactive behaviors in “3U” environments. In this vein, game theory (Fudenberg and Tirole, 1991) emerges as a pivotal tool, offering nuanced strategies and frameworks for optimizing agent-environment interactions. Concurrently, the integration of advancements in cognitive science is anticipated to play a quintessential role. Specific areas of interest encompass the optimization of information management protocols between humans and robotic agents and the augmentation of robotic cognitive faculties through the effective utilization of perception devices. These integrated approaches aim to engender a more seamless, efficient, and adaptive interaction paradigm, catalyzing enhanced performance and adaptability in complex, dynamic environments.

However, the exploration with interactive behaviors between agents and environments may be time-consuming. Involving human knowledge and value alignment in the exploration can improve its adaptability in a human-loop learning system. Future work to achieve SRRL with interactive behaviors in “3U” environments can leverage game theory (Fudenberg and Tirole, 1991) and advances in cognitive science, for instance, how to manage the information between humans and robots, and robots how to leverage perception devices to enhance its cognitive abilities.

## 5. Conclusion

Robots need to ensure safety while leveraging RL in human environments. However, conventional SRRL algorithms do not consider the mutual influence between the robot and the human. In this paper, we proposed a human-centered SRRL framework consisting of three stages: safe exploration, value alignment, and human-robot collaboration. We discussed how these stages can leverage mutual influence or bidirectional information transfer between the robot and the human through interactive behaviors. We also described four key open challenges related to the robustness, efficiency, transparency, and adaptability of SRRL for effective utilization of interactive behaviors.

## Data availability statement

The original contributions presented in the study are included in the article/supplementary material, further inquiries can be directed to the corresponding author.

## Author contributions

SG: Investigation, Methodology, Visualization, Writing—original draft, Writing—review & editing. AK: Methodology, Writing—original draft, Writing—review & editing. YD: Methodology, Writing—original draft, Writing—review & editing. GC: Supervision, Writing—review & editing. JP: Project administration, Supervision, Writing—review & editing. AK: Funding acquisition, Project administration, Resources, Supervision, Conceptualization, Writing—review & editing.
